# American Medical Association

**Published:** 1890-07

**Authors:** 


### AMERICAN MEDICAL ASSOCIATION.

(*Continued from Page 289.*)

SURGICAL SECTION—FIRST DAY, MAY 20.

President of the section, Dr. Watson, read an able paper, based on personal experiments, on Concussion of the Spinal Cord and Brain.

Discussion by Drs. Carpenter, Murphy, King, Hoadley, Coyn and Van Quast.

Dr. Merril Ricketts read a report of cases of External Surgery of the Nose, and presented photographs of his cases.

Continuous Side-stitch Suture, by Dr. C. L. Lewis, was a very interesting and instructive paper.

One of the ablest papers of the meeting was that of Dr. Lenn, on Resection of the Cæcum for Carcinoma.

SECOND DAY.

Dr. David Barrow, of Lexington, read a paper on Gunshot Wounds of the Abdomen.

Radical Operation for Hernia in Infancy and Early Childhood. A paper on this subject was read by Dr. Thomas H. Manley.

Dr. W. T. Briggs, of Nashville, presented a patient suffering from persistent piapism, which all methods of treatment had failed to relieve.

Dr. J. B. Deaver, of Philadelphia, read a paper on the Value of the Leiter Incandescent Lamp Urethroscope in the Diagnosis and Treatment of Chronic Urethral Discharges.

Dr. Howard Kelly's paper was on Supra-Vaginal Hysterectomy, or Hystero-myomectomy, with Suspension of the Stump in the Lower Angle of the Wound.

Fractures in Children, by Dr. Edward Martin, was a paper of great interest.

Dr. Robert Morris made a strong plea for peroxide of hydrogen.

THIRD DAY.

Dr. J. G. Carpenter presented a paper entitled A Plea for Early Abdominal Section in Intestinal Obstruction.

The following are the officers for the ensuing year:

Chairman—Dr. McGraw, of Detroit.

Vice-Chairman—Dr. Deaver, of Philadelphia.

Secretary—Dr. W. A. Davis.

The Management of Major Amputations, was the subject of Dr. J. G. Wyeth's paper.

Dr. Joseph Price presented a paper on Abdominal Section and Drainage for Purulent Peritonitis.

Dr. H. O. Marcy, of Boston, read a very exhaustive paper on Surgical Treatment of Biliary Obstruction. Discussed by Drs. Lenn and Deaver.

Dr. J. L. McComas read a paper on Gunshot Wound of Brain. Recovery with Ball remaining therein.

FOURTH DAY.

Dr. W. R. Townsend read a paper on the Treatment of Flat Foot by the Thomas method.

SECTION ON PRACTICE OF MATERIA MEDICA AND PHYSIOLOGY.

THIRD DAY.

Malaria and the Causation of Periodic Fevers, by Dr. H. B. Baker, of Lansing, was very interesting.

Dr. George Dock presented diagrams and read a paper on the Changes of the Blood in Malarial Fevers.

SECTION ON DISEASES OF CHILDREN—THIRD DAY.

The Significance of High Temperature in Children, by Dr. W. L. Stowell, produced a good deal of discussion by Drs. Cutter, Watson, Woodbury, Work, Brush, Larabee and Boyd.

The meeting then adjourned to listen to the discussion on Croup and Diphtheria, before the Section on Laryngology, which was very interesting, being participated in by Drs. Reiter, Porter, Jacobson, Early, Didama and Daly.

FOURTH DAY.

The following papers were read by title: Further Observations on Foot and Mouth Disease, in its Relation to Human Scarlatina, by Dr. J. W. Stickler.

Notes on a case of Tetany, by Dr. Jacob Schenck.

The Importance of Preliminary Treatment of the Nasal Mucous Membrane before Resorting to Operations, by Dr. Carl Seiler.

OBSTETRIC SECTION—THIRD DAY.

Intra-ligamentary, or Embedded Cysts, by Dr. Wathen, was a very interesting and instructive paper.

Dr. E. W. Mitchell reported two interesting cases of severe vomiting in pregnancy.

Uterus Bilocularis, was the subject of Dr. L. H. Dunning.

Fistulous Escapes of Ligatures after Abdominal Operations, was ably presented by Dr. Marie B. Werner.

Dr. A. W. Johnstone spoke of the difficulty of obtaining a good quality of milk.

Dr. Joseph Hoffman made a strong plea for the General Adoption of Traction Forceps.

A Retrospect of Pelvic Surgery, was presented by Dr. Joseph Price.

Drainage in Abdominal Surgery, was presented by Dr. G. E. Shoemaker.

Many papers were read by title in this section.

---

THE CERTAINTY OF THE DOCTORS.—"But, Doctor, you said last week that the patient would certainly die, and now he is perfectly well." "Madame, the confirmation of my prognosis is only a question of time."—*Fliegende Blœtter.*

